# Cerebral Autoregulation in Ischemic Stroke: From Pathophysiology to Clinical Concepts

**DOI:** 10.3390/brainsci11040511

**Published:** 2021-04-16

**Authors:** Ricardo C. Nogueira, Lucy Beishon, Edson Bor-Seng-Shu, Ronney B. Panerai, Thompson G. Robinson

**Affiliations:** 1 Neurology Department, School of Medicine, Hospital das Clinicas, University of São Paulo, São Paulo 01246-904, Brazil; edsonshu@hotmail.com; 2 Department of Neurology, Hospital Nove de Julho, São Paulo 01409-002, Brazil; 3 Cerebral Haemodynamics in Ageing and Stroke Medicine Research Group, Department of Cardiovascular Sciences, University of Leicester, Leicester LE2 7LX, UK; lb330@leicester.ac.uk (L.B.); rp9@leicester.ac.uk (R.B.P.); tgr2@leicester.ac.uk (T.G.R.); 4 National Institute for Health Research (NIHR) Leicester Biomedical Research Centre, University of Leicester, Leicester LE5 4PW, UK

**Keywords:** ischemic stroke, dynamic cerebral autoregulation, cerebral hemodynamics

## Abstract

Ischemic stroke (IS) is one of the most impacting diseases in the world. In the last decades, new therapies have been introduced to improve outcomes after IS, most of them aiming for recanalization of the occluded vessel. However, despite this advance, there are still a large number of patients that remain disabled. One interesting possible therapeutic approach would be interventions guided by cerebral hemodynamic parameters such as dynamic cerebral autoregulation (dCA). Supportive hemodynamic therapies aiming to optimize perfusion in the ischemic area could protect the brain and may even extend the therapeutic window for reperfusion therapies. However, the knowledge of how to implement these therapies in the complex pathophysiology of brain ischemia is challenging and still not fully understood. This comprehensive review will focus on the state of the art in this promising area with emphasis on the following aspects: (1) pathophysiology of CA in the ischemic process; (2) methodology used to evaluate CA in IS; (3) CA studies in IS patients; (4) potential non-reperfusion therapies for IS patients based on the CA concept; and (5) the impact of common IS-associated comorbidities and phenotype on CA status. The review also points to the gaps existing in the current research to be further explored in future trials.

## 1. Introduction

Stroke is a major cause of death and disability worldwide [1], with ischemic stroke (IS) accounting for 70% of all strokes [1]. Nonetheless, there are limited therapies driven to the pathophysiology of IS and most of them are mainly focused on recanalization of the occluded vessel [2,3]. These therapies targeting cerebral blood flow (CBF) restoration through the occluded vessel have proved to be efficient with high rates of recanalization and improved clinical outcomes [2,3]. Although there has been an increase in patients treated with these therapies, especially in developed countries [4], this is not the reality for many centers and not all patients are eligible for treatment due to a limited time window [2] and/or access to advanced imaging modalities and their interpretation to define a viable tissue window. Thrombolysis with recombinant tissue plasminogen activator (rtPA) is available, on average, for only 7 to 12% of patients [5] and this is reduced further for thrombectomy [6]. This remains the case in developed countries, and this number is likely to be much lower in less developed countries [7].

Furthermore, not every patient with successful recanalization has a good clinical outcome which is termed futile recanalization [8]. Different hypotheses have been put forward to explain this phenomenon, such as collateral status and/or microvascular occlusion [8,9]. However, it is also plausible to consider the role of impairment of CBF regulatory mechanisms in worsening neurological outcomes in patients with futile recanalization. Supportive hemodynamic therapies aimed at optimizing perfusion in the ischemic area could protect the brain and may even extend the therapeutic window for reperfusion therapies. However, the knowledge of how to implement these therapies in the complex pathophysiology of brain ischemia is challenging and still not fully understood. A primary example of this controversy is the lack of clear guidelines concerning blood pressure (BP) management in acute stroke, due to the lack of reliable and consistent evidence [10]. Optimal management of BP is highly dependent on the integrity of cerebral autoregulation (CA) to protect the brain from ischemia or hyperperfusion. Therefore, a promising research area is the investigation of cerebral hemodynamics and regulatory mechanisms (i.e., CA) in the early and late phases of IS, aiming to guide therapies tailored to improve cerebral hemodynamics. The aim of this review is to discuss the role of CA in IS with the objective to present the main findings of the studies, to guide the clinician/researcher to set future research towards the application of this concept into clinical practice. The authors understand that the theme is extensive, and they have selected the most relevant findings of the studies. Of note, this is not a systematic review with a detailed meta-analysis of the studies.

The main topics to be presented are:

- discuss the role of CA in the ischemic process
- the methodology used to evaluate CA in IS
- CA in IS patients
  - the evolution in different phases of IS (acute, sub-acute and chronic)
  - the relationship with stroke subtypes
  - the association with clinical outcomes
- potential non-reperfusion therapies for IS patients based on CA concept
- the impact of common IS-associated comorbidities and phenotype on CA status.

## 2. The Potential Role of CA in the Ischemic Process in the Acute Phase of IS

**Figure 1:**
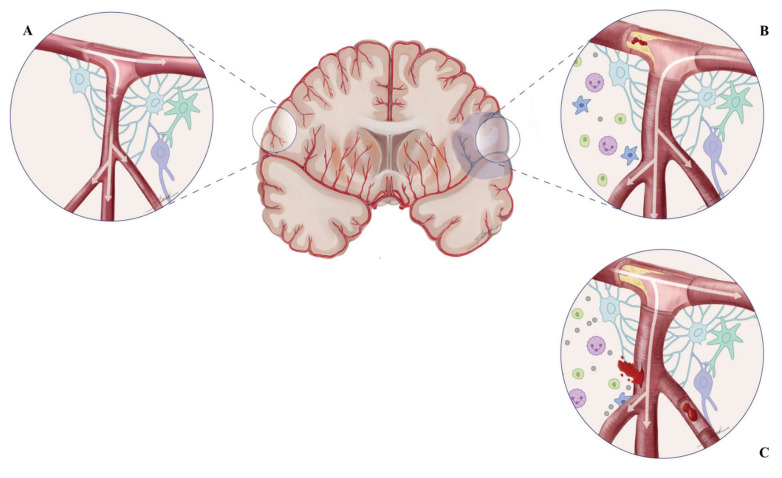
(**A**) Schematic representation of pial artery with penetrating vessel (intracerebral arteries) and its intraparenchymal components. (**B**) After vessel occlusion there is a vasodilatory response with flow diversion and inflammatory process in the ischemic area. (**C**) Perpetuation of ischemic process increases inflammatory response; at this time if the vessel is reperfused, ischemic lesions (ex. hemorrhage) and/or distal occlusion may result in futile recanalization.

Cerebral metabolic needs are highly dependent on a continuous blood supply. With blood flow interruption, brain function is impaired within seconds and irreversible damage will occur within minutes [11,12]. To prevent ischemic damage, the cerebral circulation has a number of protective mechanisms that optimize brain perfusion. The dominant mechanism that tends to maintain CBF approximately at a constant, despite wide variations in BP, is termed CA [13,14,15]. Anatomically, the CA response to changes in BP takes place at different segments of the arterial tree, involving pial arteries, intracerebral arterioles, and capillaries [11] (Figure 1A). CA is mainly affected through the myogenic response, whereby an increase in transmural pressure leads to the initial dilation of the vessel, stretching the vascular smooth muscle (VSM), leading to an influx of Ca2^+^ that induces VSM contraction and eventually a reduction in vessel diameter that restores CBF to its original level [11,16,17,18]. In addition to the myogenic mechanism, the CA response can be modulated by neurogenic pathways and metabolic control, involving CBF shear stress and the endothelial release of nitric oxide [11,18,19,20].

The primary change during IS is the interruption of blood flow within a specific territory leading to a fall in cerebral perfusion pressure and a vasodilatory response, which will be part of the collateral circulation activation response (local, leptomeningeal or global primary collaterals) which promotes flow diversion to the ischemic region [12,21] (Figure 1B). At this time, blood flow in the affected area and surrounding territories starts to reach critical flow thresholds and, despite attempts to increase CBF by collaterals and to increase oxygen extraction by cells, the affected area will begin to lose electrical activity (penumbral region) with inevitable loss of membrane function culminating in neuronal death (ischemic core) [12,22,23]. The penumbral region is the area of interest of many reperfusion therapies in acute ischemic stroke (AIS) and represents potentially viable tissue that may be salvageable depending on the duration the tissue has been underperfused, the extent of collateral supply and the integrity of CA [9,24,25,26].

In addition, the ischemic process leads to endothelial dysfunction and to a cascade of adverse consequences, such as metabolic impairment, energy failure, free radical production, excitotoxicity, loss of calcium ion homeostasis, and activation of proteases [27,28,29,30]. These adverse events increase oxidative stress and mitochondrial damage, resulting in necrosis and apoptosis-mediated neuronal cell death (Figure 1B). In this environment, if the ischemic/penumbral area is reperfused, the breakdown of the blood brain barrier function, loss of mechanisms of CA and persistent distal microvascular occlusion will contribute to reperfusion lesions (e.g., hemorrhagic transformation), and/or the lack of clinical improvement despite flow restoration (futile recanalization) [8] (Figure 1C).

Considering all the events described above, CA may represent an attractive therapeutic target to improve collateral response, maintain flow in ischemic surrounding areas, and avoid reperfusion lesions that will enhance the benefits of recanalization.

## 3. Methods Used to Assess CA in AIS

There are an extensive number of methods that can be applied to CA studies. In this section we will discuss the main aspects of the methods used in IS studies; for further information, there is literature dedicated specifically to the theme [13,14,15,31].

Assessment of CA requires methods to obtain reliable measurements of CBF and BP [13]. The duration and protocol involved in these measurements will depend on whether static or dynamic CA will be assessed [32,33]. In addition to these parameters, it is important to record continuous CO_2_ due to the high influence of this variable on CA status [31,34,35].

As AIS patients are a very critical population, any ancillary methods that can be performed at bedside will be preferred to avoid moving the patient and exposing them to instability. Given its portable nature, the transcranial Doppler ultrasound (TCD) has been extensively used in AIS patients with further advantages of excellent temporal resolution (~5 ms) [36], and relatively low cost [14,37], when compared to other imaging alternatives. TCD has some limitations as it requires specialist training with a large learning curve, some patients will have an inadequate acoustic bone window and the method relies on the assumption that changes in cerebral blood flow velocity (CBFV) are directly proportional to changes in CBF, which will be true only if the cross-sectional area of the insonated artery remains constant [38,39]. Moreover, this examination has a limited spatial resolution as it can only measure CBFV in the main intracranial vessels (MCA, middle cerebral artery; PCA, posterior cerebral artery; ACA, anterior cerebral artery), and cannot detect more localized changes in blood perfusion [39,40].

An alternative method used to study CA in AIS patients is near infrared spectroscopy (NIRS) [41]. NIRS is also a bedside method that measures the ratio of delivery and utilization of oxygen which is linked to CBF. Compared to TCD, NIRS has the advantage that is easier to install and is not limited to the bone window; but is limited by the potential contamination of the NIRS signal by the extracranial circulation and/or changes in systemic circulation, leading to erroneous interpretations [26,42]. Spatial resolution is another concern of the method, but significant advances have been made in this area [43,44]. Despite some of its potential advantages, NIRS has not been used extensively for the assessment of CA in stroke studies [37], and should be further explored.

BP monitoring is a pivotal step in CA studies [45,46,47] and although AIS are a critical population, invasive BP measurements are seldom justified. Thus, the most common method to monitor BP in this scenario is arterial volume clamping of the digital artery. One concern with this method is that non-invasive BP measurements in the finger may not be representative of the perfusion pressure in the MCA or other intracranial arteries [13,16]. However, a previous study has shown that arterial volume clamping measurements produce similar results when compared to those estimated using intra-arterial measurements in the ascending aorta [48]. Invasive BP monitoring also can be used when applicable, but usually requires a critical care setting [13].

As mentioned above, CA can be evaluated as a static or dynamic phenomenon [13,20,49,50,51]. In the AIS environment, the need for BP manipulation makes the static approach problematic and for this reason, it has been used with caution to avoid risking further lesions caused by changes in perfusion pressure [17,38,52,53,54,55]. On the other hand, dynamic CA (dCA) is expressed by the transient response of CBF to rapid changes in BP, and can be assessed over shorter periods of time. This method is more attractive for AIS patients because there is less potential for harm [31,38], including the possibility of using spontaneous BP oscillations as the stimulus to quantify the effectiveness of CA by observing the corresponding changes in CBF [13,31,56]. For this purpose, a number of different models can be used to quantify the CBF response to changes in BP [14].

By far, the most commonly used model is transfer function analysis (TFA) [13,37,57]. TFA expresses the BP–CBF relationship at each frequency that comprises the spontaneous (or induced) oscillations in BP [37]. The autoregulation index (ARI) is also widely used [14]. Although initially formulated to assess dCA with other protocols, such as the thigh cuff maneuver, not suitable for TFA [32,35,58,59], this index can also be extracted from spontaneous oscillations in BP, and it has been demonstrated to incorporate all the information in TFA, which is a significant advantage [60]. Importantly, although the use of spontaneous fluctuations in BP is very attractive in this population, there are limitations to this method, such as the degree of BP variability, that may not be sufficient to provide a reliable estimation of the dCA parameters [31] and the need to have a significant coherence, between input (BP) and output (CBF), to guarantee the reliability of estimates of gain, phase and ARI. Therefore, further developments are needed to improve the application of this method in the AIS population.

Other methods used for modeling CA are correlation coefficient indexes (e.g., Mx index, correlation of CBFV and BP; oxygen reactive index, correlation of cerebral perfusion pressure or BP and tissue oxygen) [35,41,46,61,62,63,64], rate of return [47] and multimodal pressure–flow analysis [65,66]. Project pursuit regression (PPR) is a non-linear technique that allows estimation of the flow–pressure static curve, often requiring methods such as lower-body negative pressure, to induce larger oscillations in BP [67,68] but we are not aware of its application to the assessment of AIS patients.

## 4. CA Evolution in Different Phases of IS (Acute, Sub-Acute and Chronic)

**Figure 2:**
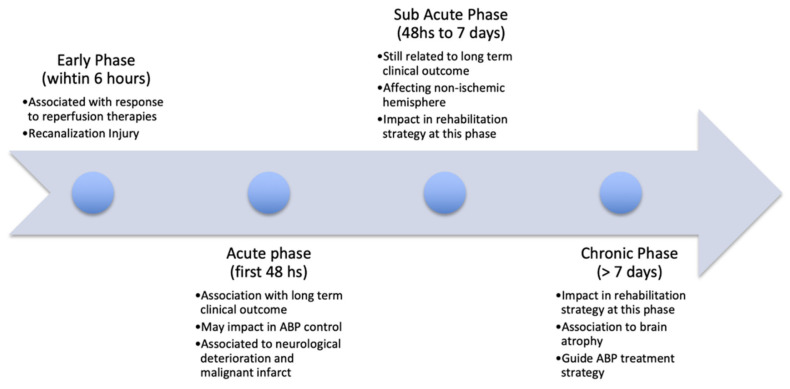
Timeline of the impact of cerebral autoregulation (CA) on ischemic stroke (IS).

Studies have found mixed results on the evolution of dCA changes in the acute (<48 h), subacute (48 h to 7 days), and chronic (>7 days) phases of IS (Figure 2). In this section we consider the evidence of dCA impairment at these major time points.

### 4.1. Acute Phase (<48 h)

There is conflicting evidence for dCA impairment in the acute phase of stroke. Reinhard et al. investigated 33 patients within 24 h of MCA infarction, and in the early phase, the dCA remained intact, as measured by phase and Mx [63]. In keeping with these findings, Lam et al. measured ARI in 15 patients at 24 h post-AIS, and did not find significant differences when compared to healthy controls [69]. In contrast to these findings, Petersen et al. found that the TFA phase shift was significantly lower in the affected hemisphere (AH) compared to the unaffected hemisphere (UH) in large vessel stroke within 48 h of onset [70]. This is in keeping with findings from studies demonstrating reduced ARI in the AH at 36 h in mild stroke [71], and 48 h in stroke of mild to moderate severity, regardless of sub-type [72]. In a study by Saeed et al., ARI was reduced within 48 h of mixed etiology stroke compared to healthy controls, but there were no differences between hemispheres [73]. Studies varied significantly in the sub-type and severity of the stroke, the dCA parameters and methods used (i.e., spontaneous TFA, thigh cuff maneuver), and whether comparisons were between healthy controls or the UH. Thus, conflicting findings may be due to methodological heterogeneity, as well as stroke population per se. In a recent systematic review and meta-analysis, the pooled results from four studies did not find a significant difference in the phase within 48 h of stroke onset [37].

### 4.2. Sub-Acute Phase (48 h to 7 Days)

In contrast to the acute phase, the majority of studies that evaluated dCA in the sub-acute phase found an impairment of CA even in the early period (48–96 h) [71,74,75,76,77] of mild to moderate AIS. Furthermore, in the later stages of the sub-acute phase (5–7 days), dCA was not just demonstrated to be impaired [55,62,63,69,71,78], but also reported to affect both the AH and UH compared to just the AH in the acute phase of large vessel stroke [55,62,63]. In contrast, Salinet et al. did not find impairment of CA in the first 72 h of AIS in a cohort of mild/moderate stroke patients [35]. Thus, differences between studies may again be attributable to methodology and choice of metrics used, in addition to infarct characteristics.

### 4.3. Chronic Phase (>7 Days)

The majority of studies have focused on the acute and subacute phases of stroke, with fewer investigating the temporal nature of changes in the chronic phase. Novak et al. found that dCA remained lower in patients more than 2 months following minor MCA infarction in the AH using head-up tilt with no impact in clinical outcomes [53]; in addition, this study observed that dCA remained preserved in the UH [53]. However, two studies found dCA changes resolved by three months post-AIS [69,79], and few studies have measured intervening time points from 7–14 days to 3 months. The most comprehensive study examined four time points post-AIS: 72 h, 14 and 30 days, and three months [79]. dCA was intact in the early (72 h) phase, reduced during the later subacute phase (14 days), but recovered at 30 days and 3 months post-stroke [79]. Similarly, Kwan et al. followed ten patients with MCA infarcts over three months, and phase shift increased over the three time-points (<7 days, 6 weeks, and 3 months) indicating improving dCA from the sub-acute to chronic phases [80]. The longest study followed patients with lacunar infarction, finding that dCA changes were sustained at 6 months after stroke onset [81]. However, this may be due to global changes resulting from small vessel disease, rather than the acute infarct [81].

### 4.4. Summary

Mixed results have been reported regarding dCA changes in the acute phase, but more consistent findings of poorer dCA at the subacute phase, subsequently recovering into the chronic phase. For some patients, dCA changes may persist into the chronic phase, particularly those with poorer functional outcomes and greater stroke severity. There was significant heterogeneity between studies in terms of the time points used, sub-types and severity of strokes enrolled, and the dCA parameters and methods used which may account for these differences, particularly within the acute phase of stroke. The evolution of these changes has important considerations for management of patients, particularly for pharmacological manipulation of BP in the acute phase (Figure 2) [61,79].

## 5. CA in Different Stroke Subtypes

To date, studies have demonstrated conflicting results on dCA impairment between stroke sub-types [54,72,73,74,77,78]. The reasons for these differences may be due to variations in the classification systems used, age, co-morbid status of the patients, stroke severity, timing (acute, subacute, chronic), and whether the diagnosis of sub-type was made clinically or with the support of neuroimaging.

### 5.1. Large and Small Vessel Artery Stroke

Dawson et al. investigated 61 patients post-AIS and found no differences in dCA between sub-types [54,55]. Similarly, studies have found no differences between cortical and sub-cortical stroke [73], or between the Oxford Community Stroke Project classification (OCSP) of stroke sub-types (TACS, total anterior circulation stroke; PACS, partial anterior circulation stroke; LACS, lacunar stroke) [72]. Xiong et al. found that dCA was lower in both hemispheres in 60 patients (30 LVO, 13 SVD, 17 mixed), in all three stroke sub-types [74]. However, patients with co-existing large vessel occlusion (LVO) and small vessel disease (SVD) pathology had the lowest dCA values of the three groups, suggesting a cumulative impact on dCA from superimposed pathology [74]. Saeed et al. also found differences in dCA between cortical and sub-cortical strokes [73]. However, the mechanism by which large vessel stroke results in bilateral impairment remains unclear. One hypothesis is that AIS results in a pro-inflammatory state which is a systemic manifestation, and thus can affect both hemispheres simultaneously [74]. Furthermore, Eames et al. suggested this may be due to loss of facilitatory axons affecting transhemispheric communication, and therefore a reduction in contralateral dCA [75].

In contrast to these studies, Imminik et al. found patients with large MCA infarcts had significantly poorer dCA in the AH only, with sparing of the UH [77]. This distinguished LVO from LACS, which were found to have more global reductions in dCA efficiency affecting both hemispheres [77]. In keeping with this finding, Guo et al. demonstrated an asymmetrical pattern of poorer dCA, selective for the AH in those with MCA stenosis (n = 15), and bilateral, non-selective impairments in small-artery stroke (n = 26) [78]. Lacunar infarcts most commonly occur in the presence of SVD, and thus reductions in dCA are more likely to reflect significant burden of cerebrovascular disease than a discrete lacunar infarction, which are typically of small volume when compared to LVO [77,78]. This is supported by the findings of a recent study of patients undergoing cardiopulmonary bypass, where SVD rather than large vessel stenosis was associated with impaired dCA [82]. In a study of stroke of undetermined etiology, the TFA phase was reduced in the UH but not the AH [83]. As this study enrolled relatively mild strokes, this may be due to an underlying mixed etiology and the presence of SVD [83].

### 5.2. Intra- and Extracranial Stenosis

Impaired dCA in the ipsilateral hemisphere has been well documented in several studies of carotid artery stenosis [84,85,86,87] and shown to correlate with the severity of stenosis [84,86]. Furthermore, dCA changes associated with carotid artery stenosis can be reversed with successful stenting or endarterectomy procedures [85,87]. Despite a clear association of altered dCA in carotid artery stenosis, the relationship with symptoms or clinical outcomes is less clear [84,88], and may be due to collateral formation [88,89]. However, in a longitudinal study of 165 patients with severe internal carotid artery stenosis, poorer dCA was associated with increased risk of ischemic stroke [90].

Similarly, studies of intracranial stenosis have reported poorer dCA in the AH ipsilateral to the stenosis [91,92,93,94], which correlates with the degree of stenosis [91,92,93,94], and is improved with stenting and collateral formation [92]. In contrast to carotid artery stenosis, dCA changes in intracranial vessels are associated with poorer clinical outcomes and greater symptomatology [92,93].

### 5.3. Anterior and Posterior Circulation

Fewer studies have investigated the effects on posterior circulation stroke, or compared anterior and posterior circulation infarcts. Gong et al. investigated dCA in the MCA and PCA in patients with basilar artery stenosis [91]. Phase shift was decreased in patients with severe stenosis compared to healthy controls and those with moderate stenosis, which was selective for the PCA [91]. Furthermore, in patients with AIS and basilar artery stenosis, lower phase shift was associated with a poorer functional outcome at 90 days [91]. Guo et al. investigated dCA changes in 71 patients with lacunar infarcts in the MCA (n = 46) and PCA (n = 25) territories [81]. Both unilateral MCA and PCA territory infarcts demonstrated bilateral reductions in phase difference, and this may be as a result of significant small vessel disease pathology causing global dCA impairment [81].

### 5.4. Summary

Taken together, the results of the dCA studies in AIS support the notion that large vessel occlusion is associated with a focal pattern of poorer dCA confined largely to the AH, whereas small vessel disease or lacunar infarctions are associated with a more global pattern of impairment affecting both hemispheres [36]. However, this finding has not been replicated across all studies, and the effects distal to the original infarct cannot be excluded. Intra- and extracranial stenoses are associated with poorer dCA, which correlate with the degree of stenosis, and is reversible with intervention. There is limited evidence comparing posterior and anterior circulation infarcts, but current evidence suggests impairments in dCA are comparable across stroke territories. The authors believe that including a wide variety of stroke classification systems with a focus on stroke mechanisms (e.g., Trial of Org 10172 in Acute Stroke Treatment, TOAST [95]) could better separate the population into groups with more homogeneous findings.

## 6. CA and Outcome in IS

Cerebral autoregulation may impact the clinical outcome of a patient with AIS (Figure 2), with possible repercussions for the effectiveness of therapeutic strategies. Furthermore, the knowledge of the time and extent of the CA impairment could guide the clinician to implement measures to preserve the vulnerable ischemic and/or reperfused area [96,97,98].

In the very early stages of AIS, more specifically, during intravenous thrombolytic therapy, dCA impairment was demonstrated to impact the response to rtPA therapy with greater improvement in NIHSS in patients with better dCA [9]. In addition, lower values of dCA metrics in the hemisphere with an ischemic lesion 24 h after thrombectomy were correlated with a clinical outcome as measured by a three-month modified Rankin scale (mRS) score [99]. These findings were corroborated by other studies [41] extending their application to a wider population that included patients not submitted to reperfusion therapies within 6 h of symptoms [96]. Moreover, it is possible that dCA impairment is not just a consequence of ischemic insult, but is also associated with other comorbidities, such as renal impairment [100]. This could explain why dCA is also impaired in the UH and correlates with chronic radiological findings of microvascular disease [100]. In addition, it has been demonstrated that dCA can influence the incidence of brain recanalization injuries, such as hemorrhagic transformation and/or reperfusion lesions (i.e., oedema) [41,101]. This finding requires further exploration because of the key impact in the management of BP in the very early phase of AIS. This would help to determine an optimal BP that is associated with better clinical outcomes and minimal reperfusion/ischemic injuries [41,96,101].

However, dCA may have a pivotal role in the clinical presentation of AIS; it was demonstrated that dCA and neurovascular coupling were not just associated with the clinical outcome measured by an mRS score after three months, but also with stroke severity at presentation [59]. Regarding the effect of severity, Dohmen et al. used invasive neuromonitoring methods to demonstrate that dCA impairment in the hemisphere affected by the ischemic insult can influence the malignant evolution of the ischemic lesion, which directly impacts the clinical outcome (three month mRS). Furthermore, dCA evolution during the sub acute phase (up to seven days) may also impact the outcome, where, in moderate-to-severe IS, there was a further deterioration in dCA with spread to the non-ischemic hemisphere, which was associated with a worse clinical outcome [62].

These findings are very important to plan the rehabilitation program post-IS. In the near future, this planning should be individualized according to multiple variables including dCA status. Moreover, with the description of patients with dCA impairment in the chronic phase of ischemic stroke (>6 months), and the correlation with functional status and brain atrophy, individualized treatments targeting CBF status (e.g., angiotensin receptor blockers) [102] may be implemented for this population [66].

There is an unexplored issue, that is, the association of dCA with post stroke dementia (PSD) [103]. It could be hypothesized that dCA is impaired in dementia, such as Alzheimer’s disease, but the findings are still controversial [104,105]. However, the consensus is that during cognitive tests, there is a change elicited by cerebral regulatory mechanisms [10,106]. Recently, a study reported a link between cognitive decline and dCA but the limited number of patients precludes definite conclusions [107]. Thus, it would be justified to investigate dCA as a potential maker of PSD.

### Summary

Dynamic CA has an impact on the clinical outcome from the very early stages of IS to the subacute and chronic stages. This is important not just to enhance the response to reperfusion therapies, but also to manage cerebral hemodynamics in the sub-acute phase to prevent malignant evolution and/or worse clinical outcomes. The authors believe that dCA may be important for planning short- and long-term therapeutic strategies, and future randomized controlled trials should be planned. There is a multicenter project called INFOMATAS (Identifying New targets For Management and Therapy in Acute Stroke) [108] that aims to prove this concept.

## 7. Potential Non-Reperfusion Therapies for IS Patients Based on CA Concept

Few studies have directly investigated the effects of therapeutic strategies for IS on dCA. Mechanistically, treatments in IS may have important effects on dCA, and monitoring hemodynamic outcomes could provide valuable prognostic and risk stratification information to guide clinical decision making. In this section we focus on the key non-reperfusion therapeutic strategies in IS that could impact dCA: BP management, hypothermia and head positioning.

### 7.1. Blood Pressure Management

There has been much debate around BP management in IS in the scientific community, and evidence from clinical trials does not support aggressive BP lowering [109,110,111,112,113]. Aggressive BP management below the lower dCA limit could risk hypoperfusion of viable ischemic penumbra, paradoxically worsening functional outcome [114,115]. On the other hand, surges in BP which breach the upper limit of CA could result in further tissue damage, worsen cerebral oedema and potentiate hemorrhagic transformation of the infarct [114,115]. Certainly, poorer dCA in the acute phase has been associated with increased risk of oedema and hemorrhagic transformation [101]. Furthermore, patients with pre-existing chronic hypertension will have adapted to a higher resting BP and will experience a rightward shift in their autoregulation curve [112,114]. The ENCHANTED trial demonstrated that intensive BP lowering (compared to the guideline) in patients who underwent thrombolysis for AIS was safe and reduced risk of intracranial hemorrhage [109]. However, this did not translate to an improvement in clinical outcomes, and the mechanistic reasons for this remain unclear [109,116]. Similarly, the ENOS trial did not demonstrate any difference in outcome in patients who continued versus those who stopped antihypertensive therapy in the acute phase [110], and the SCAST trial found increased risk of progression, and poorer functional outcome following candesartan administration in AIS [111]. Powers et al. monitored CBF during BP lowering with nicardipine infusion in nine patients post-AIS [45]. Two patients demonstrated global reductions in CBF with BP lowering, but had a rightward shift in their CA curve due to chronic hypertension [45]. These results support the notion of individualized BP goals achieved through dCA monitoring during therapy [45]. However, this was a small study, and larger sample sizes are required to investigate the effects of acute BP lowering in AIS on dCA outcomes. Given that dCA impairment is present in the acute phase and tends to worsen in the sub-acute period (Figure 2), BP management in this phase should be approached with caution in light of the findings of clinical trials [79].

### 7.2. Head-of-Bed Positioning

Positioning of the head of the bed has been investigated as a therapeutic strategy to maintain cerebral perfusion pressure and thus rescue viable penumbra in a number of studies [117]. The majority of studies that investigated this issue were small and non-randomized trials that are prone to bias [117]. The HeadPoST trial was the largest clinical trial and found no difference in the functional outcome between patients who were supine compared to seated following AIS [117,118]. The study demonstrated increased CBFv in the AH of supine compared to upright patients [117], and did not show any effects on clinical outcome or correlation between increased CBFv and improved outcome, though predominantly mild stroke patients were recruited (median NIHSS 4) [119]. Despite these findings, there is still evidence that early mobilization is correlated with clinical outcomes in more severe strokes patients [120], raising the question that head positioning could be used as an individualized strategy of treatment according to stroke severity [121].

In 39 patients with AIS, those with poorer CA had increased local cerebral blood volume with head lowering compared to an increase in those with better CA [112]. Lam et al. did not find any difference in ARI at rest or during rapid head positioning, but found a significant change of ARI during gradual head positioning in 15 patients post-AIS over three time points (<24 h, 5 days, 12 days, and three months) [69,122].

The combined results of clinical and mechanistic studies to date suggest that head position in the acute phase of stroke may have an impact on clinical or hemodynamic outcomes in AIS. In patients with poorer CA, selective head positioning may be beneficial and requires further investigation.

### 7.3. Summary

There are promising non-reperfusion therapies that could be applied in IS patients based on hemodynamic (systemic and cerebral) status. However, these therapies are still under investigation and their potential effects to dCA are speculative. The present review calls attention to the need for monitoring dCA during these prospective therapies given its potential relevance for guiding interventions.

## 8. The Impact of Comorbidities and Phenotype in CA and Stroke

The evidence base describing the relationship between co-morbidities, CA and stroke is limited. Here, we consider the main studies investigating this relationship and studies reporting dCA in common co-morbid conditions amongst the stroke population.

### 8.1. Hypertension

Chronic hypertension is the most common stroke risk factor [123], and adequate control can achieve ~30% reduction in stroke risk [124]. Furthermore, hypertension during AIS is a poor prognostic factor, with increased stroke severity and risk of poorer clinical outcomes [114,115,125]. Although studies have not demonstrated any appreciable effects on dCA [53,65,126,127,128], this does not preclude the ability of hypertension to modulate dCA in the acute phase of stroke. As discussed above, a rightward shift in the dCA curve due to physiological adaptation to chronically raised pressure in the cerebral vasculature could significantly increase the susceptibility of brain tissue to infarction in the context of acute hypoxia [112,114,129]. In two studies of hypertension and concomitant IS, ARI remained preserved due to rises in BP during the tilt [53], but the TFA phase shift was found to be reduced in hypertensive stroke patients compared to healthy controls [130]. However, these findings were not replicated in a study by Dawson et al., where dCA was unrelated to hypertension or antihypertensive treatment within 96 h or at 7 to 14 days post-event [55]. In a longitudinal study, stroke patients had sustained increases in BP at all time points (36 h, 14 and 30 days, and three months) following AIS, which may have contributed to worsening dCA in the sub-acute phase of stroke [79]. However, an effect of BP in AIS on dCA was not specifically investigated in this study [79].

Both high and low BP are considered to be implicated in cognitive decline with a link with CBF [131]. Thus, the cognitive status should be further investigated in future studies aiming to link the BP control in hypertensive patients and dCA status. Thus far, the evidence does not support aggressive BP management in the acute phase of stroke; indeed this could carry a significant risk of worsening hypoperfusion in a brain adapted to higher perfusion pressures. Future work should focus on the relationships between BP, dCA, and outcomes in AIS to develop optimal BP management strategies to guide clinicians.

### 8.2. Diabetes

Diabetes mellitus is an independent stroke risk factor [132], associated with poorer functional outcomes [133]. However, there is limited evidence to support tight glycemic control in the acute phase of stroke [134], and this is not recommended by current international guidelines [135]. Several studies have demonstrated altered dCA in patients with type 2 diabetes mellitus [97,136,137,138], and this precedes the development of microvascular complications (e.g., retinopathy, nephropathy), and autonomic dysfunction [136]. However, this is not a consistent finding, and Huq et al. did not find changes in dCA in type 2 diabetes using respiratory maneuvers [139]. No study has investigated the relationship between type 2 diabetes and dCA in the acute stroke period. However, a study investigating intensive BP lowering in patients with type 2 diabetes demonstrated that autoregulatory impairment during BP augmentation is related to the presence of microvascular complications [140]. In patients without microvascular complications, dCA was preserved with BP control, but this was lost in patients with an established microvascular disease [140]. Thus, the potential interaction between common co-morbid conditions and their treatments on dCA could be significant during the acute stroke period and warrants further investigation.

Similar to hypertension, diabetes is also linked to an increased risk of PSD [141] and the correlation of this link to dCA also should be investigated in the future. There are no consistent findings in the scientific and clinical literature to support tight glycemic control post-stroke. However, no studies have specifically examined the relationship with dCA in the acute phase which may shed additional light on whether these strategies are beneficial or harmful post-AIS.

### 8.3. Chronic Kidney Disease

Chronic kidney disease (CKD) is associated with an increased risk of AIS and is associated with severity and outcome [142,143,144]. CKD is associated with reductions in CBF and altered cerebral hemodynamics [144,145], and thus dCA impairment has been proposed as a common mechanism to both stroke and CKD that could be a potential target [142]. In a study by Castro et al. of 46 patients with AIS, a lower eGFR was associated with reduced dCA efficiency within 6 h of stroke onset, poorer functional outcome at three months, and increased risk of hemorrhagic transformation [142]. Given the similarities in cerebral and renal autoregulation, both organs are susceptible to microvascular damage from chronic conditions, such as hypertension and diabetes [142,146]. Thus, the relationship between stroke, CKD, and poor clinical outcomes may be through a shared underlying pathological mechanism of impaired dCA [142]. Given that these findings have only been investigated in one study, these require replicating with further studies in different populations to determine a consistent relationship, and whether there is an association with clinical outcomes. It remains to be determined whether optimization of renal function can modulate this apparent association between stroke, renal disease, and dCA impairment in the acute phase.

### 8.4. Heart Failure

Heart failure is an important risk factor for AIS [147,148,149], and, as with CKD, it increases the severity and odds of a poorer outcome in acute stroke [150]. Heart failure results in reduced cerebral perfusion due to lower cardiac output, but also as a result of alterations in sympathetic tone from renin-angiotensin-aldosterone system activation [150]. This produces a state of chronic cerebral hypoxia and reduced vasomotor reactivity [150,151]. In two recent studies, the efficiency of dCA was found to be reduced (ARI) in patients with heart failure [147,152]. Only one study has specifically examined heart failure in the context of AIS [150]. Patients with heart failure had a higher TFA phase compared to AIS, with intact cardiac function and healthy controls, which was associated with increased myocardial injury (higher troponin) at 6 and 24 h post-AIS, normalizing within three months [150]. These results seem to contradict previous findings of increased stroke risk, severity, and poor outcome in heart failure and AIS [148,149]. The authors postulate that this is due to ischemic preconditioning induced by chronic cerebral hypoperfusion, rendering the brain less susceptible to acute hypoxic events, such as AIS [150]. However, this was a small study with a number of limitations (bias towards aortic stenosis, confounding from antihypertensives) [150], and these results require further investigation in larger patient cohorts. Similar to those with CKD, it remains unknown whether the optimization of cardiac function with commonly used drugs (e.g., angiotensin converting enzyme inhibitors, beta-blockers), which improve outcomes in heart failure, may also modulate dCA and potentially improve outcomes in AIS.

### 8.5. Phenotype: Age & Sex

Age is a well-known risk factor for IS with a direct impact on mortality and morbidity [153,154]. Aging is also associated with increased arterial stiffness, vessel rarefaction and remodeling, all of which contribute to greater vascular resistance, higher BP and lower perfusion capability [155]. Although it has been demonstrated that with aging, there is low perfusion capability [156], there are still inconsistent results regarding dCA [157,158]. Furthermore, there is no study that correlates age with dCA in AIS patients, and this should be investigated in future studies.

Sex is another important variable in IS patients with men tending to have increased mortality and women having increased stroke severity and risk of cognitive decline [155,156]. Although there are conflicting reports of sex differences in the regulation of CBF in healthy populations [157,158], to our knowledge, this issue has not been explored in AIS patients. Although these both represent non-modifiable risk factors, understanding the relationship between age and sex, and dCA in AIS is important for prognostication and risk stratification in the acute and chronic phases.

### 8.6. Summary

Undoubtedly, comorbidities influence the hemodynamic status in patients with AIS. One of the main explanations for the impairment of CA, is the influence of co-morbid pathology prior to the ischemic event [100,146]. It remains unclear the extent to which impairments in dCA are present prior to stroke, how these are modulated by stroke, and the impact of cumulative co-morbidities on dCA in the acute and chronic phases of stroke. The question that should be further investigated is how comorbidities and demographics influence the hemodynamic cerebral response in the first hours of ischemic injury. As stroke patients have multiple comorbidities, this may be difficult to demonstrate and demands large sample sizes from multicenter trials. As mentioned in a previous section, a recent multicenter initiative (INFOMATAS) through the Cerebral Autoregulation Network has been developed to address this through a large individual patient data meta-analysis of dCA in ischemic stroke [159].

## 9. Conclusions

There have been significant advances in the knowledge of CA studies for IS patients, either in techniques for CBF monitoring or for CA modeling; the results of these advances have been translated into a recognized society guideline which will uniform the methodology of future research applied to this condition [57]. This advance has the potential to introduce the study of CA as a new target to be implemented in clinical decision-making for AIS patients. Despite the large number of studies that have been presented in this review, there is not yet enough evidence to use CA metrics to guide clinical decisions, and a multicenter trial is therefore warranted to define which CA parameters should be sought to improve patient outcomes. As highlighted in the present review, the most important variables to be included in this trial would be the (1) inclusion of different stroke subtypes; (2) different phases of IS (ultra-acute, hyper-acute, acute, subacute and chronic); (3) impact of different comorbidities on CA status; and (4) protocols to study the influence of non-reperfusion therapies (e.g., head-of-bed-positioning) to improve outcome in patients with IS. For this purpose, an international collaboration group, INFORMATAS, has been launched with the purpose to gather clinical evidence with respect to CA studies in IS, and to develop clinical trials to further explore this evidence [108]. This will be the most appropriate way to introduce CA measurement and management into routine clinical practices in IS.
